# Comment: Applications of robotics in the clinical laboratory

**DOI:** 10.1155/S1463924690000177

**Published:** 1990

**Authors:** William J. Castellani, Frederick Van Lente, David Chou

**Affiliations:** Department of Biochemistry The Cleveland Clinic Foundation 9500 Euclid Avenue Cleveland Ohio 44195 USA

## Abstract

The implementation of a robotic workstation in the clinical laboratory involves considerations and compromises common to any instrument design and development activity. The trade-off between speed and flexibility not only affects the way the instrument interacts with human operators and other devices (the ‘real-world interface’), but also places limitations on the adaptation of chemistries to the given instrument. Mechanical optimization for speed and reproducibility places restrictions on the imprecision of consumables. Attempts to adapt a robot to a constrained system may entail compromises that either degrades the theoretically-attainable quality of results, or requires human interaction to compensate for physical or mechanical limitations. The general considerations of function and workflow, programming and support, and reliability place practical limits on the implementation of robotic workstations in the clinical laboratory.


Journal of Automatic Chemistry, Vol. 12, No. 4 (July–August 1990), pp. 141–144

### Comment: Applications of robotics in the clinical laboratory

**William J. Castellani, Frederick Van Lente and David Chou**

*Department of Biochemistry, The Cleveland Clinic Foundation, 9500 Euclid Avenue, Cleveland, Ohio 44195, USA*

*The implementation of a robotic workstation in the clinical laboratory involves considerations and compromises common to any instrument design and development activity. The trade-off between speed and flexibility not only affects the way the instrument interacts with human operators and other devices (the 'real-world interface'), but also places limitations on the adaptation of chemistries to the given instrument. Mechanical optimization for speed and reproducibility places restrictions on the imprecision of consumables. Attempts to adapt a robot to a constrained system may entail compromises that either degrades the theoretically-attainable quality of results, or requires human interaction to compensate for physical or mechanical limitations. The general considerations of function and workflow, programming and support, and reliability place practical limits on the implementation of robotic workstations in the clinical laboratory.*

### Introduction

Bunce *et al.* [1] reviewed the current status of laboratory robots and provided provisional specifications for a clinical laboratory robot. The authors' work [2] with an early model of the Zymate system (Zymark Corp. Inc., Hopkinton, Massachusetts, USA) has led them to several conclusions about the role of such robots in clinical chemistry laboratories, which provide a useful addition to the previously cited paper.

Three issues are essential considerations in implementing a robotic workstation: proposed function and integration into workflow; programming and support; and reliability. These systems are currently investigational; turn-key systems of general applicability do not exist. Therefore, the laboratory must be ready to deal with all the issues of developing and maintaining a unique production system.

The authors' workstation [2] was designed to accept from one to 24 patient samples, take three aliquots from each, and perform immunologically-based sample preparation for cardiac isoenzymes using the Roche Isomune-CK and LD kits (Roche Diagnostics, Nutley, New Jersey, USA). In brief, immunoprecipitation using an antibody against the lactate dehydrogenase M subunit isolates the LD1 fraction from one aliquot of patient sample. A second aliquot is treated with excess antibody against creatine kinase M subunit, blocking all activity from this subunit; immunoprecipitation is used to remove all CK-M containing isoenzymes from the third aliquot. CK2 (CK-MB) activity is equal to twice the difference between the activities in the second and third aliquots. The robot would perform all pipetting and centrifugation steps, including placing the completed samples in cups for analysis on a Cobas-BIO (Roche Analytical Systems, Nutley, New Jersey, USA). Human interaction was limited to placement of the untreated patient samples on the workstation, replenishing reagent and consumables, and transferring the completed samples to the BIO for analysis. The robot disposed of reaction tubes and used pipette tips in containers for later removal.

It is important to distinguish between the highly automated ('robotic') multipurpose/multichannel analysers common in the clinical laboratory and general-purpose robots ('robotic workstations'). On a basic level, the trade-off between the two systems is speed versus flexibility. Automated analysers place rigid constraints on the tasks that can be performed and on the interface with the outside world. For example, if a chemistry cannot be adapted to the limitations of the instrument (timing constraints, measurement limitations, sample-to-reagent ratios, etc.), it just cannot be done on the analyser. A given analyser may take its final 'endpoint' measurement on a chemistry well before equilibrium, simply because the instrument is incapable of running a reaction long enough for it to reach equilibrium. If the chemistry is not linear to concentration under non-equilibrium conditions, the implementation may well be impossible. Secondly, the machine–world interface is strictly controlled; specimens and reagents must be placed on the instrument properly for the system to work. Sample preparation must be done off the machine, and often the data must be corrected for any dilutional effects by the operator after analysis. This was the greatest limitation in fully automating the immunochemical procedures for cardiac isoenzymes described in this article: there simply is no way for a general-purpose robot to place a sample holder and reagents on a multichannel analyser and to press the 'start' button – a human interface must exist. Such rigidity in the machine-world interface is tolerable because of the extreme adaptability of the human operator, who must conform to the analyser's limitations. This allows the analyser to be tailored to its task, permitting range-of-motion and instrument layout to be optimized for speed. In contrast, a general-purpose robot, by definition, must be adaptable to the task in hand, requiring the ability to handle liquids, solids, and gases; to carry, aliquot, weigh and measure; and to conform to the layout of the workbench. This flexibility requires a greater sophistication in spatial awareness: the robot must be able to go from any point in its large work envelope to any other point, by some route, while still avoiding any obstacles. Speed can be obtained only by increasing the sophistication of the robot – and, therefore, its cost and complexity – or by decreasing the number of tasks it can perform (until you reach the point of having a 'specialized robot' – as typified by the

0142–0453/90 $3.00 © 1990 Taylor & Francis Ltd.

multichannel automated analysers described above). At present, the cost/benefit ratio between speed and cost/complexity favours slower robots.

### Function and workflow

The primary consideration in implementing a robotic workstation is the selection of appropriate tasks for the robot to perform; this supersedes consideration of what robot system to purchase, since attempting to automate an inappropriate task will make even the best robotic workstation into a 'boondoggle'. There are basically two types of tasks that appear amenable to implementation: long, tedious procedures that are not time-critical, and short, rapid-turnaround procedures that are done 'stat' with small batch sizes. Many industrial systems have been described based on the former criterion [3] and often perform sample preparation for high-pressure liquid or gas chromatography, spectrophotometry, or radio- and enzyme immunoassay. Two characteristics of these procedures are that turnaround time is slow due to the nature of the tasks (requiring repeated incubations and separations), and that the ultimate determinant of workflow – the 'bottleneck' – is usually the analyser (HPLC, GC, gamma counter, etc.) that is used in the final step, typically handling only one specimen at a time. Automation of these procedures has three benefits: it frees an individual who would otherwise be occupied in long, tedious tasks; it maintains consistent performance characteristics, given the high reproducibility of robotic motions and workflow; and it allows potentially hazardous samples and reagents to be handled in isolation. The systems that have been described are not turn-key workstations; they were designed, programmed, and maintained by a limited number of 'specialists', often employees at the laboratory. Most clinical laboratories do not have the resources to implement such a system, no matter how beneficial and cost-effective it might be.

The authors' work, on the other hand, was directed towards automating a procedure that was performed 'stat' in their laboratory. The cardiothoracic surgeons at the Cleveland Clinic Foundation monitor open heart surgery patients, during the immediate 16–24 hour post-operative period, with three determinations of cardiac-derived isoenzymes; the immediate post-operative and the 8 hour post-operative specimens are drawn and analysed throughout the day effectively on a stat basis. The procedure as implemented on the authors' robotic workstation represented a change in methodology (immunochemical rather than electrophoretic), with a shorter turnaround time; the workflow, however, would be comprised of multiple small batches performed at irregular times throughout a 16 hour work period. The robot became the limiting factor with large batch sizes – a batch of 24 samples required 3 hours to complete, versus approximately 45 min when done by a technologist. However, the application presupposed small batch sizes, where the robot throughput was comparable to human performance. With small batch sizes, 'non-robotic' steps (incubation, centrifugation, filtration, etc.) are the main time determinants of run time, and impact equally on both robotic and human throughput. True stat procedures are eminently automatable on robotic systems; however, cost-effectiveness becomes a significance issue. These procedures tend to be sporadic and low volume; high-volume stat procedures are ready targets for automated multichannel analysers, and instrument manufacturers will attempt to implement such procedures if at all possible. A robotic workstation will be cost-effective only if the peculiar requirements of an institution makes a particular stat test into a high volume procedure, or if several such low-volume procedures can be offered on the same robot. The attractiveness of general-purpose robots is that this becomes possible; however, these robots must be programmed and maintained in-house for multiple procedures, requiring significant dedication of developmental resources before the robotic workstation can become cost-effective. The 'functionality' of the system is also important. Whitney has discussed the design of 'a robot to wash a stack of dishes' [4]. When confronted with this problem, beginning students conceive a mechanism that reproduces a human's actions in manipulating, inspecting and cleansing a plate. He then described the standard automatic dishwasher as a possible solution. These are not equivalent. An automatic dishwasher will not deal with a stack of dishes on a countertop. It will not ensure cleanliness. It demands much greater human involvement. In a similar manner, a robotic diluter/dispenser (Tecan Robotic Sample Processor RSP 505, Tecan US, Chapel Hill, North Carolina, USA) was programmed to perform the aliquotting and reagent addition steps. This device was much faster than the robotic workstation in processing larger runs; however, the implementation was only partial, since a technologist had to centrifuge and decant the sample preparations. Speed was optimized by limiting the functionality of the automated device.

The specific requirements, the 'robustness', of a given procedure is important in determining programming constraints, and, ultimately, throughput. With the procedure for cardiac isoenzymes, the robot was faster than a technologist in processing a batch size of three or less; this was an unintended outcome of the programming. Each patient sample was divided into three aliquots, to be processed separately; one aliquot was to be incubated for 20 min with an antiserum, then analysed. The technologist and the robot both prepared this aliquot first, performed all the necessary manipulations on the remaining specimens, then submitted the first aliquot for analysis. However, the technologist waited the requisite 20 min incubating time; the robot did not wait – and with a run of a single patient sample, this aliquot would be submitted for analysis after 17 min, completing the entire procedure faster than the technologist by cheating on this incubation time. This did not affect the validity of the analytical result, so this program feature was acceptable. Deviations from the recommended procedure may be pronounced when comparing a patient sample run singly rather than as part of a batch; the same aliquot that took 17 min to complete when part of a single-specimen run would incubate for 3 hours if it was the last one in a batch of 24. The robustness of the procedure allowed for considerable flexibility in programming incubation times; otherwise, the robot would have to be freed from other activities at the end of critical incubation times for the procedure to work correctly. The only practical way to do this is to have the robot stop all activities just prior to these times and wait. This places severe constraints on the programming, which in most instances would result in even longer run times. The ingenuity of the programmer is then challenged; the procedure can be implemented, certainly, but is it worth the effort?

### Programming and support

Given that laboratory robotic workstations are currently task- and laboratory-specific, the programming and support of such systems must be done in-house, perhaps with initial assistance from the robot manufacturer. The authors' workstation took approximately 300 hours of time to bring up and evaluate, almost evenly divided between three general tasks: initial program development; evaluation and optimization of the program modules; and assessing the precision and accuracy of the completed system – it did not include the initial evaluation of the task, the selection of necessary peripheral devices, and the in-house manufacture of pieces of equipment that were necessary but were not available from outside sources. The initial development included the laying out of the workbench, the programming of locations and motions, and the integration of modules of activity into a smoothly-functioning whole. The workbench layout was in fact broken down and re-configured during this phase of the programming; it was thought essential to be willing to backtrack as far as necessary to correct inefficiencies that became apparent with time. It is all too easy to accept a module or a benchtop layout once it is established, no matter how detrimental it proves to be to program the system around its inadequacies. Although we have separated program development time from evaluation and optimization, these activities usually occur concurrently. It is difficult to write a given program module without having established that the robot can successfully execute all the preceding steps and the programmer must know in what state and location the robot, samples, and reagents will be on entering the program module. The final phase, that of testing and documenting the procedure, encompasses the routine evaluation that a clinical laboratory performs when establishing a new test method, and needs no further comment.

### Reliability

This section is more theoretical than the above discussions, which were based on actual experience. Although reliability issues arose and were dealt with during the testing and evaluation of the laboratory robotic workstation, the system never went into production and the reliability issues of a stat device operating throughout the day were never evaluated – this was a result of decisions external to the robotic implementation, and not due to any difficulty with the implementation. Nonetheless, three general principles can be stated: 'kludges' are often marginal at best; consumables will get you every time; and reliability takes time.

A 'kludge' has been defined as 'an assembly of ill-assorted parts forming a distressing whole' [5], and may best be described as the predecessor to a prototype. One factor distinguishes a kludge: it works. It may be unreliable, it may be inefficient, but it does work. A robotic workstation assembled from manufacturer-provided and tested equipment may be quite reliable as a prototype; it becomes a kludge when the users begin modifying the original equipment, or adding parts that are built in-house or appropriated from other devices. For example, the authors modified a 6-in. sampling cannula to act as a meniscus detector to permit direct aspiration from patient sample tube; electronically, the device worked with admirable reliability. However, rather than buying and mounting the cannula on a second hand, we chose to manipulate it by grippers on our general-purpose hand that were originally designed for carrying test tubes; inconsistencies in the grasp of the cannula allowed for considerable variation in the position of the cannula tip. On occasion, this caused the cannula to miss the opening of a sample tube and hit the surface of the test-tube rack. The resultant 90° bend in the cannula caused significant problems. However, with experience, it was possible to bend the cannula back to approximately the same shape that it had before the accident, so that it was serviceable without rebuilding it or reprogramming the relevant positions in the robot. Several program steps were inserted to secure the cannula in the grasp of the fingers, which improved reliability during the evaluation of the system; nonetheless, manipulation of this cannula remains as one of two major weak points in the system. The temptation to add to or modify peripheral devices is strong, since this may lead to increased speed, often at little monetary cost; however, compromised reliability may well be a consequence of this effort.

A second concern is the reproducibility of the physical characteristics of the consumables. As an example, the robot had no problems in attaching, using and shucking the disposable pipette tips that were provided with the system. However, the system developed persistent pipetting problems when tips were purchased directly from the original manufacturer, even though the same part number was used. The manufacturer had made a special mold for the tips supplied with the robot which had thicker walls than the standard tips, but the same part number as for the standard tips was used. These standard tips did not sit in the holding rack reproducibly. This problem was solved by simply running a hand over the rack, aligning the tips in a standard position. The robot manufacturer was also able to re-machine the pipette tip rack to fit the standard, less-expensive tips. Nonetheless, certain locations in the pipette rack were unusable due to high failure rates when the robot attempted to access standard tips in these positions. The manufacturing imprecision and lot-to-lot variation that is acceptable in a typical bench-top situation becomes totally unacceptable when applied to a blind robot. This is the second major weak point in the system.

The final consideration is the degree of reliability that the system must provide. Given that a simple procedure was being automated, it was felt that sporadic run failures could be tolerated, with either a re-start of the program or manual execution of the run. However, if the failure rate proved to be too high, or if the procedure had to be more reliable, then function checks would have to be implemented throughout the program. The system could pass test-tubes through light beams or press buttons with attached pipette tips to make sure that the test-tubes were picked up and that the pipette tips were attached – typical points of failure. However, such checks take time and tie up the robot arm; the authors were willing to leave these steps out and accept a low level of sample or run failures as a result. As a global issue, a complex system composed of several electromechanical devices will have a failure rate for each device and for the entire system. It is difficult, at best, to replace a failed piece of equipment with a non-identical substitute; therefore, failure of almost any part will bring the entire system to a halt unless an identical spare is available. No significant failures were experienced during evaluation of the system, but it is impossible to evaluate this in production – especially with wear of the equipment and changes in tolerances. If failure is not tolerable, then the system must incorporate check stations, and spare devices must be kept on hand – and it cannot be proved at this time that current laboratory robotic workstations ever reach the reliability of the human technologist over the long term.

### Conclusion

General-purpose laboratory robots have proven themselves in industrial applications, both in manufacturing and quality control, and typically in tasks that are long, tedious and not dependent on rapid throughput. Laboratory robots may also have a niche in low-volume, rapid-throughput situations such as stat testing. Their highly-reproducible actions eliminate the concern of maintaining adequate bench skills on a technically-demanding procedure that is done rarely; their 24 hour stand-by nature decreases the level of round-the-clock staffing to cover for the sporadic sample that may come through at any time. However, the lack of turn-key systems that can be brought in as complete, operational devices precludes their use in most clinical laboratories at present. A concerted effort to develop, implement and maintain a robotic workstation must be made to obtain any benefit – requiring a significant allocation of resources, at least initially. The concerns described herein are not unique to robotic workstations and would need to be resolved in any design and development protocol. The one question that cannot be addressed is long-term reliability – which imparts a significant uncertainty to any cost/benefit assessment. However, appropriate tasks can currently be automated on such devices with a reasonable expectation of success and savings in human manpower; each potential user must decide whether he has the programming and financial resources to attempt such a venture.

### References

1. Bunce, R. A., Broughton, P. M. G., Browning, D. M., Gibbons, J. E. C. and Kricka, L. J., *Journal of Automatic Chemistry*, **11** (1989), 64.
2. Castellani, W. J., Van Lente, F. and Chou, D., *Clinical Chemistry*, **32** (1986), 1672.
3. Hawk, G. L. and Strimaitis, J. R. (eds), *Advances in Laboratory Automation – Robotics* (Zymark Corp., 1984 onwards annually).
4. Whitney, D. E., *Harvard Business Review*, **86** (1986), 110.
5. Granholm, J. W., *Datamation*, **8** (1962), 30.

